# Supplementary material for: Development of the Rabbit NASH Model Resembling Human NASH and Atherosclerosis
Source: Biomedicines. 2023 Jan 27;11(2):384. doi: 10.3390/biomedicines11020384 (PMC9953079; doi:10.3390/biomedicines11020384)

**Supporting information**

**Supplementary Table 1. Nutrient composition of the control diet (LRC4).**

・**Nutrient composition (per 100 g of diet)**

| | | **unit** |
|---|---|---|
| Water | 8.5 ± 0.3 | g |
| Crude protein | 18.4 ± 0.3 | g |
| Crude fat | 3.0 ± 0.1 | g |
| Crude ash | 8.3 ± 0.4 | g |
| Crude fiber | 16.7 ± 0.7 | g |
| Nitrogen free extract (NFE) | 45.1 ± 0.7 | g |
| Energy | 280.6 ± 2.5 | kcal |
| | | |
| Vitamin A | 303 ± 139 | IU |
| Vitamin D3 | 89 ± 43 | IU |
| Vitamin E | 6.5 ± 0.5 | mg |
| Vitamin $B_1$ | 0.76 ± 0.07 | mg |
| Vitamin $B_2$ | 1.29 ± 0.11 | mg |
| Vitamin C | 58 ± 8 | mg |
| | | |
| Calcium | 1.14 ± 0.06 | g |
| Phosphorus | 0.52 ± 0.03 | g |
| Magnesium | 0.31 ± 0.03 | g |
| Sodium | 0.19 ± 0.05 | g |
| Potassium | 1.64 ± 0.12 | g |

**Supplementary Table 2. Primer sequence used for real-time PCR**

| Gene | Accession number | Primer sequence (5’- 3’) |
|---|---|---|
| GAPDH | NM_001082253 | F 5’-GGCAAAGTGGATGTTGTCGC-3' |
| | | R 5’-GATCTCGCTCCTGGAAGATGG-3' |
| ABCA1 | XM_008257418.2 | F 5’-GGCGCTGACTCAAGATGTCTCT-3' |
| | | R 5’-TACGGCAGCACATAGGTCAG-3' |
| ABCB11 | NM_001082083.1 | F 5’-GAGGGAACTACCAGGACAGTTTAAGA-3' |
| | | R 5’-TGCCAGGTAAGAAAGCTGAGACT-3' |
| ABCG5 | XM_008254466.2 | F 5’-TTGAGTGCGCCTACAAGGAA-3' |
| | | R 5’-CACCGCCTGCTTATTCCTCA-3' |
| ABCG8 | XM_017340804.1 | F 5’-TGTCACCTCCAAATGCCACTC-3' |
| | | R 5’-GACAATGTAGGCGCAGTGCT-3' |
| ACACA | XM_008271162.2 | F 5’-ACCATTAGCCTGGTGACGTG-3' |
| | | R 5’-CCCAGCGCCTGTGAGAATTA-3' |
| ACADL | XM_002712562.3 | F 5’-GACAAATCGTGAAGCTCGCTC-3' |
| | | R 5’-GCTGGCAACCGTACATCTTC-3' |
| ACADM | XM_002715911.3 | F 5’-TTGGGTTGACTGAACAGCAG-3' |
| | | R 5’-AGGGACAGGGTATTCACCAG-3' |
| ACAT2 | XM_002714983.3 | F 5’-CAATGGCAGGTGAGTCGAGA-3' |
| | | R 5’-TCACTTCGGTAGGCCCTTTC-3' |
| ACOX1 | XM_008250924.2 | F 5’-CCGGGCAGCCAGATTAGTAG-3' |
| | | R 5’-GCTCGAACAAGGTCCACAGA-3' |
| ACSL1 | XM_008250560.2 | F 5’-ACAAGTGGAACCACAGGCAA-3' |
| | | R 5’-TGAGGCTTAGGGCCAATCAA-3' |
| ACTA2 | NM_001101682.2 | F 5’-CCCACAACGTGCCCATCTAT-3' |
| | | R 5’-CACGTTCAGCAGTAGTCACG-3' |
| APOB | XM_008254783.2 | F 5’-TCGACGTAGGCCATATGCAG-3' |
| | | R 5’-TGCTGCTCCTAGCAAAGTCC-3' |
| ASC | XM_002721750.3 | F 5’-GGGATCAAGGCCCATTCTGA-3' |
| | | R 5’-GCTGAAGAGCTTCCTCATCTTG-3' |
| BIM | XM_017340061.1 | F 5’-CGGATCGGAGACGAGTTCAA-3' |
| | | R 5’-AATGCATCCTCCACACCAGG-3' |
| CASP1 | XM_008262043.2 | F 5’-ACTGCCAGAAGGTAGGACAC-3' |
| | | R 5’-GATAGCACTCTTGGCTTCGTAT-3' |
| CAT | XM_002709045.3 | F 5’-GCTGAGAAGCCTAAGAACGC-3' |

| | | |
|---|---|---|
| | | R 5’-TGGAAAGCTGGGTGAGCATC-3' |
| CCL2 | NM_001082294.1 | F 5’-GCTGTGATCTTCATGACCAAACT-3' |
| | | R 5’-CGTGTGTTCTTGGGTTGTGG-3' |
| CD14 | NM_001082195.2 | F 5’-CCATCAATTATGGAGCCCGT-3' |
| | | R 5’-AAGTTGCAGACACAGCGGAT-3' |
| CD36 | XM_008258299.2 | F 5’-TGCTAGACATCGGCAAGTGT-3' |
| | | R 5’-AGCCGCTTTGCAAACTGTAA-3' |
| CD68 | XM_008270775.2 | F 5’-TCCCAAGAGAGGGAAGAGGG-3' |
| | | R 5’-CCCTGGGCTGCGTGTAG-3' |
| COL1A1 | XM_017348831.1 | F 5’-TAAAGGGTCACCGTGGCTTC-3' |
| | | R 5’-GAGGCCGTTGAGTCCATCTT-3' |
| COL3A1 | XM_002712333.3 | F 5’-CCACTCGGACTTGCAGGAAT-3' |
| | | R 5’-GCTTCCCACTTTCACCCTTG-3' |
| CPT1 | XM_008252852.2 | F 5’-TTAACAAACTGTGTCCTGTGGC-3' |
| | | R 5’-AGCTTCAGCGATTGCCCAGA-3' |
| CTGF | XM_017345378.1 | F 5’-AACATTAAGAAGGGCAAGAAGTGC-3' |
| | | R 5’-ATCGGCCATCGGTGCATAC-3' |
| CYBB | NM_001082100.1 | F 5’-GGATGAGTCCCAGGCCAATC-3' |
| | | R 5’-TTCAGCCAAGGCTTCAGGTC-3' |
| CYP27A1 | NM_001190430.1 | F 5’-CTCACGTGGGCCCTATACCA-3' |
| | | R 5’-ACACAAACTGGGTGTTCTTGG-3' |
| CYP2E1 | XM_002718772.3 | F 5’-AGTACTCCTTGGCTGCAGGT-3' |
| | | R 5’-TCCGTGCTGTGTTTGTCCTTC-3' |
| CYP7A1 | NM_001170929.1 | F 5’-TGACATGCCAGTACTAGACAGC-3' |
| | | R 5’-AGGAACCATCCTCAAGGTGC-3' |
| CYP7B1 | XM_008255573.2 | F 5’-CCTGGTCTACCTAGAAAGCGTC-3' |
| | | R 5’-AGGCAGAAGTTCCCGGTTTC-3' |
| CYP8B1 | NM_001082622.1 | F 5’-ATACACTTGGCCAGCACCAA-3' |
| | | R 5’-TCAGGTAGCCAGCCTTGAAC-3' |
| DDIT3 | XM_017350485.1 | F 5’-ACACCTGAAAGCAGAACTGAG-3' |
| | | R 5’-CCTCCTGCAAGTCCTCATACC-3' |
| DGAT1 | XM_008253884.1 | F 5’-GTGTGGTGATGCTGATCCTGA-3' |
| | | R 5’-CAGGCGTTTCTCAACCTGGA-3' |
| DGAT2 | XM_008263828.2 | F 5’-TTCCTCGTGCTAGGAGTGGC-3' |
| | | R 5’-TTTCTTGGGCGTGTTCCAGT-3' |
| FABP1 | XM_002709637.3 | F 5’-CGGGAAGCACTTCAAGCTCA-3' |

| | | |
|---|---|---|
| | | R 5’-CAGCTTCACCACTGTCTTGACC-3' |
| FASN | KF201292.1 | F 5’-CAACTACGGCTTTGCCAACTC-3' |
| | | R 5’-CGATGACCGTGTCGTTGGTG-3' |
| FDFT1 | XM_008248858.2 | F 5’-GTGTGTGTTGTCTGCCCTCA-3' |
| | | R 5’-ATACCGCATGGCGCATTTC-3' |
| HMGCR | XM_017339036.1 | F 5’-GGGTATTGCTGGCCTCTTCA-3' |
| | | R 5’-AGCTTCATTCAAGCCCGTCA-3' |
| HMGCS1 | XM_008262167.2 | F 5’-AATGAGCAGGCATCCACAGAG-3' |
| | | R 5’-CAGGCATGGTGAAAGAGCTGTG-3' |
| HMOX1 | XM_002711415.3 | F 5’-CCGAGGGTTTTAAGCTGGT-3' |
| | | R 5’-TAGACCGGGTTCTCCTTGTTG-3' |
| HTGL | NM_001082032.1 | F 5’-ACAAAGCCTAAGACCAGAGCCAT-3' |
| | | R 5’-ATGGAGCCGAATCTGACAGC-3' |
| IL-1β | NM_001082201.1 | F 5’-TCTGCAACACCTGGGATGAC-3' |
| | | R 5’-CATGGAGAACACCACTTGTTGG-3' |
| IL-18 | NM_001122940.1 | F 5’-GAACCAGAAGAGGCTTGCAT-3' |
| | | R 5’-TCTGATTCCAGGTTCTCATCG-3' |
| LDLR | NM_001278865.1 | F 5’-AGCAACCGCATCTACTGGTC-3' |
| | | R 5’-TGCTGATGACGGTGTCGTAG-3' |
| MDR1 | NM_001082159.1 | F 5’-TACAGAAAGTGAGAAGGTGGTC-3' |
| | | R 5’-ACAAGTATCTCCCATCTCCCA-3' |
| MFE2 | XM_008254935.2 | F 5’-GCAGCTTGTTTGAGGTTGGA-3' |
| | | R 5’-GGCTTGCTGGCATTATCGAAG-3' |
| MRP2 | XM_008270326.2 | F 5’-ACCGGAATTTGGAACCCACA-3' |
| | | R 5’-ATATGCAGTGGTGCCCTTGA-3' |
| MTTP | XM_002716956.3 | F 5’-AGCTCCCTTCAGGCGATTTG-3' |
| | | R 5’-GCAAATACCGCCTTGCACAT-3' |
| MYD88 | XM_002723869.3 | F 5’-TGTCTCTCGACTCTTGGAACTG-3' |
| | | R 5’-CTCAGCTTTGCTACGAGAACA-3' |
| NCEH1 | XM_008266500.2 | F 5’-GCGCACAGCAAGTGAGTAAC-3' |
| | | R 5’-CCTGGCTTGAGAGAGCCTTC-3' |
| NCF1 | NM_001082102.2 | F 5’-AGTGAGAGCACCAAGCGGAAG-3' |
| | | R 5’-CCTCTGTCCTCATTCTGGAAACA-3' |
| NLRP3 | XM_017339176.1 | F 5’-ACCTCAACAAGCGCTACACA-3' |
| | | R 5’-CCAGGATGGTCTTCCCGATG-3' |
| NQO1 | XM_002711667.3 | F 5’-GAAGGACATCACAGGCAAGCTG-3' |

| | | |
|---|---|---|
| | | R 5’-CCTTCAGAATGGCAGGGACTC-3' |
| NPC1L1 | NM_001082228.1 | F 5’-TTCCACAAGTACCTGCCCTG-3' |
| | | R 5’-TGAAGCGGGTGGCTACAATC-3' |
| NTCP | NM_001082768.1 | F 5’-GACAAGGTGCCCTATGGAGG-3' |
| | | R 5’-AGAGGACAGCAACCGCTATG-3' |
| OATP1B3 | XM_008259567.2 | F 5’-AGTCTAAGTCCAGGTGATCGT-3' |
| | | R 5’-TCAGTGCGAGAGCTACCAAG-3' |
| PERK | XM_017340230.1 | F 5’-CAGACGAGTTCGTCCGAGAG-3' |
| | | R 5’-CGCAGGACACAGTTCAAAGC-3' |
| PUMA | XM_017338809.1 | F 5’-ATGGGACTTCTGCCCTTACC-3' |
| | | R 5’-GCTGGGAGTCCCGTATGCTA-3' |
| SOD1 | NM_001082627.2 | F 5’-ACCATCCACTTCGAGCAGAAG-3' |
| | | R 5’-GTCCTGTTATGCGTCCCTTGA-3' |
| SOD2 | XM_008263745.2 | F 5’-CAACGGTGGAGGTCACATCA-3' |
| | | R 5’-AGCAGTGGAATAAGGCCTTTGG-3' |
| SQLE | XM_017341522.1 | F 5’-TTCTGCCACAGATGATTCCCT-3' |
| | | R 5’-GCAACAGCAAAGAAGTGTCCA-3' |
| SR-BI | NM_001082788.1 | F 5’-TTGCCGAGATGAACGACTCC-3' |
| | | R 5’-TCGGAGTGCCAGAAGTTCAC-3' |
| SREBP2 | XM_002721384.3 | F 5’-GACGCCAAGATGCACAAGTC-3' |
| | | R 5’-TAGGTCGATGCCCTTCAGGA-3' |
| TGFβ | XM_008249704.2 | F 5’-ACTGCTTCAGCTCCACAGAG-3' |
| | | R 5’-CAGGACCTTGCTGTACTGGG-3' |
| TLR2 | NM_001082781.1 | F 5’-CGCTGAAAAACCTGACCGAC-3' |
| | | R 5’-TGTGTATCCGTGTGCTGGAC-3' |
| TLR4 | NM_001082732.2 | F 5’-TGTTCACAGAAAATGCCAGGA-3' |
| | | R 5’-GTAATATTGGGAACGACCTCCAC-3' |
| TNFα | NM_001082263.1 | F 5’-GCCCACGTAGTAGCAAACCC-3' |
| | | R 5’-GTGAGTGAGGAGCACGTAGG-3' |
| VLDLR | NM_001082188.1 | F 5’-CTCAGTGCATCCCAGTGTCC-3' |
| | | R 5’-CTGCACGTTACATTGCCACAG-3' |

**Supplementary Table 3. Primary antibodies for western blotting.**

| Protein name | Abbreviation | Manufacturer | Catalog no. |
|---|---|---|---|
| 4-hydroxy-nonenal | 4-HNE | JaICA (Shizuoka, Japan) | MHN-100P |
| glyceraldehyde-3-phosphate dehydrogenase | GAPDH | GeneTex (Hsinchu City, Taiwan) | GTX28245 |

**Supplementary Figure 1. Full-length blots of 4-HNE.**

The bands within the boxed area were used in the analysis.

The band intensity was quantified and demonstrated in Fig. 6C.

・**4-HNE**

Control HFHCD

4W 8W 14W

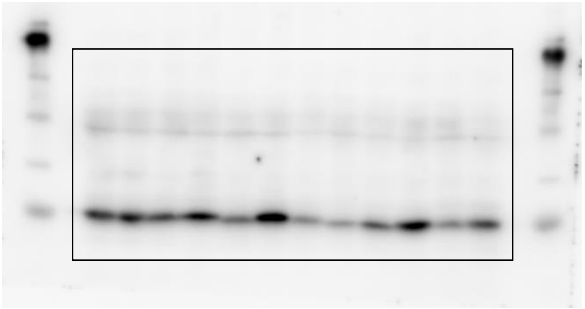

・**GAPDH**

Control HFHCD

4W 8W 14W

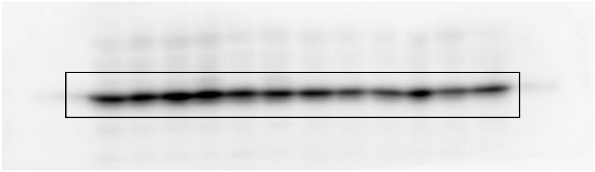

Supplement: Supplementary file 1 [file biomedicines-11-00384-s001.zip › biomedicines-2141935-supplementary.pdf]
